# SUBSTANCE USE AND ABUSE IN NEW HAMPSHIRE, MISSISSIPPI, AND WYOMING: FINDINGS FROM THE HEALTHY AGING DATA REPORTS

**DOI:** 10.1093/geroni/igae098.2524

**Published:** 2024-12-31

**Authors:** Alexa Fleet, Josephine Boateng, Megan Siebecker, Elizabeth Dugan

**Affiliations:** University of Massachusetts Boston-- Gerontology, Boston, Massachusetts, United States; University of Massachusetts Boston, Dorchester, Massachusetts, United States; University of Massachusetts Boston, Boston, Massachusetts, United States; University of Massachusetts Boston, Boston, Massachusetts, United States

## Abstract

Addiction issues in older adults are prevalent, yet often undetected or untreated, and can contribute to poor physical health, increased disability, and earlier mortality. The current study describes state rates of substance use indicators of older adults 65+ in NH, MI, and WY, and drug overdose deaths for all ages. These three states were chosen due to the high percentages of older adults residing in rural areas. The data sources are the Behavioral Risk Factor Surveillance System (2013-2020) and the Medicare Current Beneficiary Summary File (2018). Small area estimation techniques were used to calculate age-sex-adjusted community rates for more than 150 health indicators. This research examines disparities in rates for three substance use and abuse-related indicators: substance (drug and alcohol) use disorder, tobacco use disorder, and opioid deaths. Substance use disorders rates among 65+: MI 3.9%, NH 5.5%, and WY 2.4%. Tobacco Use Disorder or Current Smoker among adults 65+: MI 13.1%, NH 10.4%, and WY 11.1%. Drug overdose deaths (all ages): MI n=2,137, NH n=1,279, and WY n=1,225. Results showed variability in rates across states. MI had the highest community rates of tobacco use/smoking rates and drug overdose deaths. NH had the lowest rates for smoking but the highest rates for substance use disorders. Wyoming had the lowest substance use disorder rates and lowest drug overdose deaths. Understanding the distribution of rates makes disparities evident and may aid practitioners and policymakers in allocating resources to areas of highest need.

